# Validation of the French Clinical Assessment Interview for Negative Symptoms in a Sample of Stable French Individuals With Schizophrenia

**DOI:** 10.3389/fpsyt.2022.836600

**Published:** 2022-04-01

**Authors:** Yasmine Laraki, Cindy Lebrun, Marine Merenciano, Margot Eisenblaetter, Jerôme Attal, Alexandra Macgregor, Amandine Decombe, Delphine Capdevielle, Stéphane Raffard

**Affiliations:** ^1^University Department of Adult Psychiatry, CHU Montpellier, University of Montpellier, Hôpital la Colombière, Montpellier, France; ^2^Université Paul Valéry Montpellier 3, EPSYLON EA, Montpellier, France; ^3^IGF, University of Montpellier, CNRS, INSERM, Montpellier, France

**Keywords:** negative symptoms, schizophrenia, rating scale, validation, Clinical Assessment Interview for Negative Symptoms

## Abstract

**Objectives:**

The Clinical Assessment Interview for Negative Symptoms (CAINS) is an interview-based instrument evaluating the existence and severity of negative symptoms in people diagnosed with schizophrenia or schizoaffective disorder. The aim of this study is to translate and validate a French version of the CAINS in a French sample of outpatients diagnosed with schizophrenia or schizoaffective disorder.

**Methods:**

In this study, we included 84 outpatients with a diagnosis of schizophrenia from the University Department of Adult Psychiatry in Montpellier, France. All participants were assessed for the severity of negative symptoms as well as level of depression. Psychometric properties of the French CAINS were investigated including its factor structure, internal consistency, and interrater and test–retest reliabilities. We also determined the discriminant and convergent validity.

**Results:**

Exploratory factor analysis and parallel analysis reproduced the two-factor model, and explained 43.55% of the total score variation with good internal consistency (Cronbach α of 0.87). Both interrater and test–retest reliabilities were high for the CAINS and its subscales (intraclass correlation coefficient range, 0.89–0.99). The standard errors of measurement and minimal detectable change were also investigated. Convergent validity of the CAINS was underpinned by correlations obtained with various measures of negative symptoms. Adequate discriminant validity was established by showing that the CAINS did not correlate with positive symptoms.

**Conclusion:**

Overall, our results obtained were similar to those found in the original study of the CAINS. Structural analyses also replicated the two-factor model of the CAINS. Our results indicate that the French CAINS has robust psychometric properties and is a valid tool for evaluating negative symptoms in French-speaking individuals diagnosed with schizophrenia.

## Introduction

Researchers and clinicians have consistently shown that negative symptoms of schizophrenia are resistant to pharmaceutical treatments and persist even when positive symptoms are in remission (1–3). Moreover negative symptoms are associated with augmented caregiver burden, lowered prognostic outcomes, and poorer social functioning and may even be a predictor of social impairment within this population (4–8). A great deal of research has attempted to better understand the underlying mechanisms and constructs of negative symptoms, with the objective of increasing treatment options for this population. Increased understanding of negative symptoms requires the development of assessment tools that specifically capture the underlying constructs of negative symptoms, such as stated by the National Institute of Mental Health (NIMH) (9).

Historically, the most frequently used assessments to evaluate negative symptoms were the Scale for the Assessment of Negative Symptoms (SANS) (10) and the negative subscale of the Positive and Negative Syndrome Scale (PANSS) (11). Although the PANSS and SANS are often used in both clinical and research settings, it has been shown that caution is necessary in the interpretability of the results obtained using such scales (see for review) (12, 13). Evidence shows that some items included in these scales (such as attentional impairment or mannerisms) do not actually reflect negative symptom domains of schizophrenia (9, 14). Following the NIMH conference, experts concluded that negative symptoms are best conceptualized through five negative symptom domains (anhedonia, asociality, avolition, alogia, and blunted affect) and that rating scales should focus on findings drawn from affective sciences and fields of psychopathology (9). Recently, the European Psychiatric Association (EPA) have presented specific recommendations on how to better assess and obtain a more comprehensive understanding of negative symptoms present in individuals diagnosed with schizophrenia (14). As such, the EPA encourages clinicians and researchers to use second-generation scales such as the Brief Negative Symptom Scale (BNSS) (15) and the Clinical Assessment Interview for Negative Symptoms (CAINS) (16). Overall, these instruments share several similarities, such as having undergone rigorous analytical procedures, containing 13 items, including comprehensive manuals and probing interview questions and cover the five core negative symptom domains (17). Large-scale validation studies have also demonstrated strong psychometric properties for the BNSS and the CAINS (see for review) (14, 17). Hence, validations of the BNSS and CAINS are paramount in improving the assessments and treatment of negative symptoms in people with schizophrenia (9, 14).

To this date, the BNSS has been translated and validated into the French language in a large European and multicenter study (18) and has undergone cross-cultural psychometric analyses (19), but validation of the CAINS in the French language has yet to be done.

The main objective of the creators of the CAINS was to create a reliable, practical, and comprehensive tool that would best measure negative symptoms and would serve in both clinical and research settings (20). Following rigorous and multilevel analytic techniques including item deletions, modifications, and revisions, the developers of the CAINS were able to produce a clear, psychometrically valid scale that best captures the underlying mechanisms of negative symptoms (4, 5, 16, 20). This tool is a semistructured scale that measures constructs of motivation, pleasure, interest for social relationships and affective expression (5). Contrarily to the BNSS, the CAINS contains a lengthier interview that can provide more of a nuanced analysis of the range and frequencies of pleasurable activities (17). Overall, the CAINS measures negative symptoms across three life domains: recreational, social, and vocational. The CAINS includes assessments in terms of behavioral involvement in activities and experiences in terms of motivation, thus allowing for a complete image of the negative symptomatology (4). The validation of the original CAINS included nearly 500 individuals diagnosed with schizophrenia (5, 16), As the CAINS has become a common tool used to measure negative symptoms in schizophrenia and has been translated and validated in German, Spanish, Korean, Serbian, and Chinese, consistently showing high psychometric properties throughout (21–25).

The aim of the present study was to translate the original English version of the CAINS into French and evaluate its factor structure, internal consistency, and both interrater and test–retest reliability. We also determined the discriminant and convergent validity of this French version of the CAINS.

## Materials and Methods

### Participants

A total of 84 stable outpatients with a diagnosis of schizophrenia or schizoaffective disorder were recruited from the University Department of Adult Psychiatry in Montpellier, France. All participants had to be aged between 18 and 60 years, have a confirmed diagnosis of schizophrenia based on the *Diagnostic Statistical Manual of Mental Disorders, Fifth Edition* (26), and were able to speak and understand French. Patients were excluded from participating if they had a history of a traumatic brain injury or if they were unable to participate in the assessment (e.g., if there was presence of severe psychiatric symptoms). All participants included in this study were considered to be in stable phases of illness by the investigator, which was defined by no hospitalizations over the past 6 months, and no changes in pharmacological treatments were expected within the month of recruitment (or within the month preceding recruitment).

This study was approved by the institution’s ethics committee (IRB approval number: 202100974) and is in line with the Declaration of Helsinki 1975 standards for human experimentation.

### Procedure

The CAINS and its corresponding manual was translated and adapted into French following the specific guidelines for translations and cultural adaptation standards (27). To begin, we contacted the initial developers of the CAINS and obtained formal authorization to conduct the French translation and adaptation of the tool. The CAINS was then translated into the target language by two independent professional translators. A third expert then compared both forward translations and resolved any discrepancies between both versions into a single target translation. A back-translation was then done by a bilingual expert; a comparison between the back-translation and the original tool was revised, and any discrepancies were discussed and resolved. A panel of experts then compared the back-translation to the original version in order to verify that both English versions were equivalent in terms of conceptual content. Following a panel discussion, a harmonized version of the tool in the target language was proposed. A pretest was conducted on 10 patients in order to verify that the interview was understandable by our target population. Given that no difficulties were observed, a finalized version of the tool was proofread and any remaining errors were corrected before obtaining the final version of the French CAINS.

Once the finalized version of the French CAINS was developed, the two psychologists received full training on administering and scoring the CAINS. The training took place at the University Department of Adult Psychiatry in Montpellier and included videotaped interviews as well as group discussions regarding the scoring. Specifically, the training covered how to administer the evaluation, based on the probing questions of the manual, as well as how to conduct the ratings, based on the training videos and gold standards provided by the developers of the CAINS (5, 12, 16, 20). Gold standard ratings of the videotaped interviews were also provided, which allowed the research team to increase rater competency. The trained research psychologists had previously received full training regarding the other measures used in this study [PANSS, SANS, and Calgary Depression Scale for Schizophrenia (CDSS)].

Once recruitment for this study was ready to begin, patients were contacted to participate in this study. All participants received a full explanation of the study and its objectives before giving their informed written consent. Participant inclusions began with collecting sociodemographic data and treatment dosages before administering the different clinical assessments. Interrater reliability was possible with the assistance of the second trained research psychologist who scored simultaneously the CAINS assessment for 22 participants. For the test–retest reliability, the same research psychologist assessed another 22 participants twice (at a 2-week interval).

### Measures

The CAINS (5) is a 13-item interview-based instrument evaluating the existence and severity of negative symptoms in people diagnosed with schizophrenia or schizoaffective disorder (4, 20). The semistructured interview includes specific questions and prompts for the clinician administering the test. Scoring is based on specific descriptors provided for each item and their corresponding ratings (16). All items are scored on a 5-point scale ranging from absent/no deficit (0) to severe deficit (4), whereby higher scores reflect greater impairment. The CAINS comprises two subscales: motivation and pleasure (MAP; nine items) and expression (EXP; four items). Generally speaking, the MAP subscale evaluates the presence of decreased motivation for close relationships and in experienced pleasure. The EXP subscale measures deficits in terms of expression of emotions and speech. Subscale scores as well as total CAINS scores may be calculated by adding the results of each item. The time frame of the CAINS questions refers to experiences over the past week.

The PANSS (11) was used to determine convergent and discriminant validities of the French CAINS. The PANSS is a 30-item scale used to assess severity of symptomatology in individuals diagnosed with schizophrenia. Similarly to the CAINS, the PANSS measures symptomatology over the last 7 days. The PANSS comprises three subscales: positive, negative, and general psychopathology symptoms. Every item is rated on a 7-point scale ranging from 1 (absent) to 7 (extreme). Higher scores on the PANSS reflect greater impairment. The PANSS has been translated and validated in French with good psychometric properties (28). In this study, the PANSS had high internal consistency (total score, α = 0.87) (29).

The SANS (10) was used to measure the convergent validity of the French CAINS. The SANS is a clinician-rated scale composed of 25 items grouped into the following five domains: (1) affective flattening, (2) alogia, (3) avolition–apathy, (4) anhedonia–asociality, and (5) attention. All items are scored on a 6-point scale ranging from 0 (absent) to 5 (severe), whereby higher total scores represent greater impairment. The SANS has been translated and validated into French (30) with good psychometric properties. In this study, the SANS had excellent internal consistency (total score, α = 0.94) (29).

The CDSS (31) was used to measure the level of depressive symptoms. The CDSS is a structured interview scale composed of nine items. The total score of the CDSS can be calculated by adding the scores of each item. Each item can be scored on a 4-point scale ranging from 0 (absent) to 3 (severe), again higher scores indicating greater level of depression. The French version of the CDSS (32) has good internal consistency as well as good interrater reliability. In this study, this scale had acceptable internal consistency (total score, α = 0.77) (29).

### Statistical Analysis Plan

All variables were tested for normality prior to analysis, and no normal distribution was considered if absolute values for skewness and kurtosis were greater than 3 and 10, respectively (33). First, we ran preliminary analyses concerning the demographic and clinical variables.

Item and scale characteristics [means, standard deviations (SDs), and corrected-item correlations] of the CAINS and its subscales were calculated. Internal scale consistency of the CAINS was calculated using means of Cronbach α (34). Values between 0.81 and 0.90 indicate good reliability, and values great than 0.91 are considered excellent (29).

Test–retest reliability and interrater reliability of the CAINS were evaluated using the intraclass correlation coefficient (ICC) with a 95% confidence interval (35). ICC was based on a two-way (random-effects) repeated-measures analysis of variance model with absolute agreement. Generally speaking, ICC results are considered good if the ICC is greater than 0.7, and anything greater than 0.9 is considered of excellent reliability (29). In order to differentiate between actual difference and random measurement error, we utilized the standard error of measurement (SEM) and minimal detectable change (MDC), considered best practice in clinical literature (36). The SEM [SD × √(1 − ICC)] was calculated for the CAINS and subscales for both test–retest and interrater reliabilities. We created a variable of SD difference by calculating the difference between the SDs of the first test scores and retest scores. The SEM was then used to calculate the MDC with 95% confidence using the following formula: MDC = SEM × 1.96. The SEM is an estimation of the expected random variations in scores when no real change has occurred (36). The MDC uses the SEM to estimate the minimal amount of change that needs to be perceived, for a real change to be considered, and would not be due to inherent variation (36, 37). For a clearer clinical understanding, the MDC may be expressed as a percentage (MDC%), representing a relative amount of random variation of measurement. The MDC% can be calculated using the following formula: (MDC/mean of the CAINS) × 100 (37).

In order to investigate the CAINS structure and establish construct validity an exploratory factor analysis (principal axis factoring method with an oblimin rotation) was performed. The Kaiser–Meyer–Olkin measure of sampling adequacy (KMO) and Bartlett test of sphericity were first performed to measure whether our sample was large enough to conduct an exploratory factor analysis. KMO values between 0.70 and 0.79, between 0.80 and 0.90, and greater than 0.90 are good, mediocre, and superb, respectively. Given that the Kaiser criterion for factor extraction (eigenvalues >1.0) (38) may cause an overestimation of the number of factors (39), we decided to use scree plot and a parallel analysis (40) set at 0.01 to determine the factors to be retained.

Pearson correlations between the CAINS and clinical variables were used to determine both convergent and discriminant validities. Specifically, convergent validity was established by examining whether the CAINS subscales (and individual items) significantly correlated with negative symptoms (based on the SANS and PANSS negative subscales). Discriminant validity was shown by verifying that the CAINS did not correlate with positive symptoms (PANSS positive subscale). A Bonferroni correction was applied to correct for multiple comparisons (set at *p*_corr_ ≤ 0.003).

All analyses were performed using the Statistical Package for the Social Sciences, version 24.0, with a two-tailed α level of 5% (41).

## Results

Descriptive analyses were carried out using descriptive statistics. Data normality was confirmed for all variables using skewness and kurtosis standards (33). Demographic and clinical variables for the 84 participants are presented in Table 1.

**TABLE 1:** Demographic and clinical characteristics (*N* = 84).

	Value [mean ± SD (range) or %]
**Demographic variables**	
Gender (female)	23%
Age, years	35.50 ± 9.1 (19–59)
**Clinical variables**	
Age at onset of illness, years	23.00 ± 5.78 (14–43)
Duration of illness, years	12.32 ± 8.59 (0.25–8.59)
Antipsychotic treatment (atypical, typical, clozapine, combination)	61.0, 8.5, 25.6, 3.7%
PANSS total score	59.76 ± 16.08 (36–138)
PANSS positive	13.30 ± 5.64 (7–40)
PANSS negative	16.44 ± 6.13 (7–37)
PANSS general psychopathology	30.02 ± 7.62 (19–61)
SANS total score	40.07 ± 21.66 (2–114)
Flat affect	10.30 ± 6.95 (0–31)
Alogia	4.13 ± 4.16 (0–19)
Apathy	5.7 ± 3.66 (0–15)
Anhedonia	8.43 ± 4.56 (0–19)
Attention	2.29 ± 2.48 (0–9)
CDSS total score	4.24 ± 3.87 (0–16)

*PANSS, Positive and Negative Syndrome Scale; SANS, Scale for Assessment of Negative Symptoms; CDSS, Calgary Depression Scale for Schizophrenia.*

### Item, Subscale Analyses, and Internal Consistency for the French Clinical Assessment Interview for Negative Symptoms

Item and subscale analyses for the French version of the CAINS are presented in Table 2. All corrected item-total correlation values for the 13 items were higher than 0.3, ranging from 0.41 to 0.69, with a mean of 0.54, indicating that items correlated well with the subscales (29). The correlation between the MAP and the EXP subscales was moderate (*r* = 0.47, *N* = 84; *p* ≤ 0.01). Internal consistency reliability was high for the total CAINS scale (α = 0.87), as well as for the MAP (α = 0.86) and EXP (α = 0.82) subscales.

**TABLE 2:** Internal consistency of CAINS item and subscale scores (*n* = 84).

	Mean	SD	*r*	Scale α
Motivation and pleasure subscale score	17.05	7.02		0.855
Item 1: social, family relationships	1.02	0.99	0.53	
Item 2: social, friendships	1.52	1.06	0.49	
Item 3: social, past week pleasure	1.54	1.18	0.51	
Item 4: social, expected pleasure	2.19	1.12	0.67	
Item 5: vocational, motivation	2.33	1.29	0.52	
Item 6: vocational, expected pleasure	2.99	1.09	0.41	
Item 7: recreation, motivation	1.70	1.27	0.59	
Item 8: recreation, past week pleasure	1.74	1.12	0.69	
Item 9: recreation, expected pleasure	2.01	1.17	0.61	
Expression subscale score	5.30	3.71		0.819
Item 10: expression, facial	1.69	1.13	0.47	
Item 11: expression, vocal prosody	1.29	1.16	0.57	
Item 12: expression, gestures	1.46	1.21	0.51	
Item 13: expression, speech	0.86	1.11	0.51	
CAINS total score	22.35	9.35		0.871

*CAINS, Clinical Assessment Interview for Negative Symptoms; SD, standard deviation. r, corrected item-total correlation; Scale α, Cronbach α.*

*All items were scored on a 5-point scale (0–4), with a higher score indicating greater impairment.*

### Test–Retest Reliability

The ICC for the test–retest reliability of the total score of the CAINS was excellent: 0.92 with a 95% confidence interval ranging from 0.82 to 0.97; *p* ≤ 0.0001. The SEM and MDC were, respectively, 1.92 and 3.76, for the CAINS total scores, indicating that a change of approximately 4 points on the CAINS represents a real change with a 95% confidence level. The MDC% was 13.81%, signifying that a change of 13% in the CAINS total score is appropriate to detect a real change in symptomatology in an individual with a 95% confidence. Precisely, the average ICC for the CAINS MAP subscale was 0.89, ranging from 0.74 to 0.95 (*p* ≤ 0.0001). For the MAP subscale, the SEM was 1.97, and the MDC was 3.85. Concerning the EXP subscale of the CAINS, the average ICC was 0.89, ranging from 0.74 to 0.96 (*p* ≤ 0.0001). The SEM and MDC for the EXP subscale were 1.45 and 2.84, respectively.

### Interrater Reliability

The ICC for the total CAINS score was 0.99 (*p* ≤ 0.0001), with a 95% confidence interval ranging from 0.96 to 0.99, signifying high interrater reliability for the CAINS total scores. The SEM and MDC were, respectively, 1.23 and 2.42 for the CAINS total scores with an MDC% of 12.05%. The average ICCs for the MAP and EXP subscales were 0.99 and 0.96, respectively. Concerning the MAP subscale, the SEM was 0.82, and the MDC was 1.61. The SEM and MDC for the EXP subscale were 0.78 and 1.53, respectively.

### Construct Validity

**FIGURE 1:**
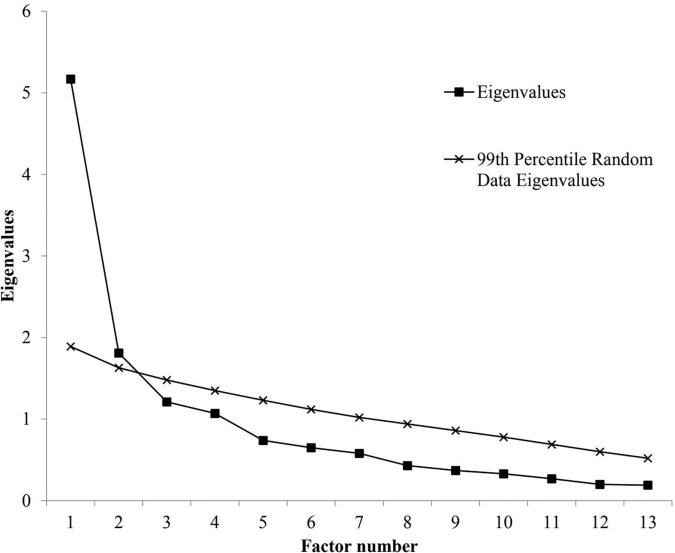
Scree plot and parallel analysis of eigenvalues for the Clinical Assessment Interview for Negative Symptoms (13 items) factors.

The KMO (KMO = 0.80) and Bartlett test of sphericity (Bartlett χ^2^ = 495.26, *p* ≤ 0.001) indicate that the CAINS was psychometrically fit for exploratory factor analysis. Examination of the scree plot and parallel analysis suggested a two-factor solution (Figure 1). Each extracted factor from the exploratory factor analysis exceeded the corresponding mean random data set eigenvalue at the 99th percentile (factor 1, 5.17 vs. 1.86; factor 2, 1.80 vs. 1.62). The next factors were not supported (factor 3, 1.20 vs. 1.48; factor 4, 1.06 vs. 1.32; factor 5, 0.73 vs. 1.22; factor 6, 0.64 vs. 1.11; factor 7, 0.58 vs. 1.03; factor 8, 0.42 vs. 0.93; factor 9, 0.37 vs. 0.85; factor 10, 0.32 vs. 0.77; factor 11, 0.26 vs. 0.69; factor 12, 0.19 vs. 0.60; and factor 13, 0.18, vs. 0.50). Based on these criteria, a forced two-factor exploratory factor analysis using principal axis factoring with oblimin rotation was performed on the CAINS. The eigenvalues were 4.67 and 1.38 explaining 35.93 and 10.62%, respectively. Factor 1 loadings ranged from 0.32 to 0.87. The loadings for factor 2 ranged from 0.50 to 0.85. Table 3 indicates the different loadings for each item on the two factors. Factor 1 regroups items 1–9 and similarly to the original version of the CAINS represents the motivation and pleasure subscale. Factor 2 regroups items 10–13 and refers to the expression subscale.

**TABLE 3:** Exploratory factor analysis for the French CAINS items with OBLIMIN rotation.

CAINS items	Factor 1	Factor 2
Item 8: recreation, past week pleasure	0.87	
Item 9: recreation, expected pleasure	0.83	
Item 7: recreation, motivation	0.79	
Item 3: social, past week pleasure	0.65	
Item 4: social, expected pleasure	0.62	
Item 1: social, family relationships	0.50	
Item 2: social, friendships	0.45	
Item 6: vocational, expected pleasure	0.41	
Item 5: vocational, motivation	0.32	
Item 12: expression, gestures		0.85
Item 11: expression, vocal prosody		0.81
Item 10: expression, facial		0.71
Item 13: expression, speech		0.50

*CAINS, Clinical Assessment Interview for Negative Symptoms.*

### Convergent and Discriminant Validities

The clinical indicators used to test convergent and discriminant validity are presented in Table 4. Bonferroni correction for the correlations resulted in lowering the required *p*-values for significance level set to *p*_corr_ ≤ 0.003. For the convergent validity, the CAINS scale was strongly associated with the PANSS negative subscale (*r* = 0.71), as well as with the SANS total score (*r* = 0.73). In addition, we further demonstrated convergent validity by testing the correlations between similar items of the CAINS with the similar subscales of the SANS, that is, of alogia, blunted affect, anhedonia, and apathy. We obtained positive and significant correlations for all of the symptoms; alogia, *r*(83) = 0.58, *p* < 0.001; blunted affect, *r*(83) = 0.68, *p* < 0.001; anhedonia, *r*(83) = 0.65, *p* < 0.001; and apathy, *r*(83) = 0.60, *p* < 0.001. These conclusive results provide additional support toward convergent validity of the CAINS. The CAINS was not related to the positive subscale of the PANSS, nor with depression (measured using the CDSS), indicating discriminant validity. Only the CAINS EXP subscale was moderately associated with the general psychopathology subscale of the PANSS.

**TABLE 4:** Convergent and discriminant validity of the CAINS.

	CAINS motivation/pleasure subscale	CAINS expression subscale
**PANSS**		
Negative subscale	0.60*	0.65*
Positive subscale	0.02	0.14
General psychopathology subscale	0.27	0.34*
**SANS**		
Blunted affect subscore	0.43*	0.72*
Alogia subscore	0.28	0.50*
Anhedonia/asociality subscore	0.70*	0.54*
Apathy/avolition subscore	0.60*	0.47*
Attention subscore	0.14	0.35*
**CDSS**	0.24	0.15

*CAINS, Clinical Assessment Interview for Negative Symptoms; PANSS, Positive and Negative Syndrome Scale; SANS, Scale for the Assessment of Negative Symptoms; CDSS, Calgary Depression Scale for Schizophrenia.*

*Pearson correlations with Bonferroni correction resulted in lowering of the p-value level set at p_corr_ ≤ 0.003.

## Discussion

This study validated a French version of the CAINS in a sample of 84 patients diagnosed with either schizophrenia or schizoaffective disorder. The mean scores in our sample ranged from 0.86 to 2.99, reflecting a moderately symptomatic sample. The internal consistency values of the overall French CAINS and its subscales were high. We obtained similar Cronbach α compared with those obtained in the original tool (5): CAINS (0.76), MAP (0.74), and EXP (0.88) subscales and to other language versions of the scale (21–24). The interrater and test–retest reliabilities were also high with ICC values >0.9, showing excellent reliability over time and across assessors. Overall, and as mentioned by authors of other validations of the CAINS (5, 21–24), we obtained very high reliabilities indicating that the accompanying manual of the CAINS is straightforward, comprehensive, and helpful in obtaining similar results across raters and time. Furthermore, the MDC obtained indicates that when using the French CAINS to measure negative symptomatology, a change in total score of 4 points shows a true change at a 95% confidence level. Item-total correlation values were also high, ranging from 0.41 to 0.69, indicating very good discrimination between the 13 items. Additionally, structural analyses of the French CAINS reproduced the two dimensional structure found in the original development of the tool, encompassing a motivation/pleasure and an expression dimension (4, 5, 16, 20). The item pertaining to motivation in the vocational domain had a relatively low loading unto factor 1 (0.32) compared with the other loadings. This may indicate that this item is difficult to measure within this population as participants may be motivated in engaging in vocation related and/or school activities but do not actually initiate any active engagements within these domains. Similarly to the initial validation (16) and in the final validation (5) of the CAINS, this item had low loadings into the MAP subscale (0.43 and 0.24, respectively). Ultimately, the low loadings of this item onto the MAP subscale may indicate that this variable has a weak influence unto the motivation and pleasure factor.

As expected, both subscales of the French CAINS highly correlated with the negative subscale of the PANSS demonstrating strong convergent validity. Similar correlations were found in our study compared with the original validation of the CAINS (5). Interestingly the motivation/pleasure subscale mostly correlated with the anhedonia and apathy dimensions of the SANS, which was also the case in the original validation. The strongest correlation with the expression subscale was with the flat affect dimension of the SANS, and this was also found in the original validation of the tool. Furthermore, no significant association was found between the alogia subscore and the MAP subscale of the CAINS. Kring et al. (5) also showed no association between these two variables. The attention dimension of the SANS was not evaluated in the original study (5); however, our results indicate an association between attention and the EXP subscale of the CAINS.

Discriminant validity of the CAINS was good and showed thorough non-significant correlations with the positive subscale of the PANSS. Similarly to results of the original validation of the CAINS (5), no correlation was found between the CAINS and level of depression. Hence, the French version of the CAINS seems to measure conceptually distinct symptoms than those of depression and positive symptoms of psychosis.

Our study included a few limitations. Although our sample size was large enough to conduct factor analyses, we did not have a 10:1 ratio between the number of participants and number of items in the scale. Another limitation is that both interrater agreement and test–retest were assessed only in a subsample of the group. An additional limit to this study is regarding the relatively low symptomatology severity of our sample group. Indeed, all participants involved in the study were in stable phases of illness and under pharmacological treatments. Future validations would benefit from including participants with a larger variation in positive and negative symptomatology. Finally, even though our study reproduced the two-factor structure of the original CAINS, a confirmatory factor analysis on another schizophrenic population would further validate the structure of this tool in the French language.

## Conclusion

Validity and reliability of the French CAINS were high and comparable to the findings described in the original version of the CAINS (5). Based on the results obtained in this study, the French CAINS has robust psychometric properties and is a valid assessment tool that can be used to evaluate the motivational and expressive mechanisms of negative symptoms in French-speaking individuals diagnosed with schizophrenia. The CAINS is a tool that was developed to address negative symptoms based on affective neuroscience research, in large samples, and using robust data analyses approaches. Further validations and cross-cultural psychometric analyses would be necessary to further validate this second-generation rating scale. This would provide clinicians and researchers another tool, in addition to the BNSS, for valid negative symptom assessment options, with hopes of ultimately bettering our care of people with schizophrenia. The French CAINS evaluates the five negative symptom domains across different life areas and is psychometrically valid and consistent with the current literature of the negative symptoms, hence a useful tool that can be used in both clinical and research environments.

## Data Availability Statement

The raw data supporting the conclusions of this article will be made available by the authors, without undue reservation.

## Ethics Statement

The studies involving human participants were reviewed and approved by the Institutional Review Board, Montpellier University Hospital, approval number: 202100974. The patients/participants provided their written informed consent to participate in this study.

## Author Contributions

SR and DC designed the study. JA, AM, AD, and ME participated in the recruitment process. YL and MM recruited and collected the data. YL, CL, and SR analyzed and interpreted the data. All authors contributed to the manuscript.

## Conflict of Interest

The authors declare that the research was conducted in the absence of any commercial or financial relationships that could be construed as a potential conflict of interest.

## Publisher’s Note

All claims expressed in this article are solely those of the authors and do not necessarily represent those of their affiliated organizations, or those of the publisher, the editors and the reviewers. Any product that may be evaluated in this article, or claim that may be made by its manufacturer, is not guaranteed or endorsed by the publisher.
